# Social Media Users’ Perception of Telemedicine and mHealth in China: Exploratory Study

**DOI:** 10.2196/mhealth.7623

**Published:** 2018-09-25

**Authors:** Ricky Leung, Huibin Guo, Xuan Pan

**Affiliations:** 1 Department of Health Policy, Management and Behavior School of Public Health University at Albany, State University of New York Rensselaer, NY United States; 2 School of Economics and Management Hebei University of Economics and Business Shijiazhuang China; 3 Department of Economics and Finance School of Economics and Management Tongji University Shanghai China

**Keywords:** mHealth, telemedicine, China, social media, text mining, keyword analysis, mobile phone

## Abstract

**Background:**

The use of telemedicine and mHealth has increased rapidly in the People’s Republic of China. While telemedicine and mHealth have great potential, wide adoption of this technology depends on how patients, health care providers, and other stakeholders in the Chinese health sector perceive and accept the technology.

**Objective:**

To explore this issue, we aimed to examine a social media platform with a dedicated focus on health information technology and informatics in China. Our goal is to utilize the findings to support further research.

**Methods:**

In this exploratory study, we selected a social media platform—HC3i.cn—to examine the perception of telemedicine and mHealth in China. We performed keyword analysis and analyzed the prevalence and term frequency–inverse document frequency of keywords in the selected social media platform; furthermore, we performed qualitative analysis.

**Results:**

We organized the most prominent 16 keywords from 571 threads into 8 themes: (1) Question versus Answer; (2) Hospital versus Clinic; (3) Market versus Company; (4) Doctor versus Nurse; (5) Family versus Patient; (6) iPad versus Tablet; (7) System versus App; and (8) Security versus Caregiving. Social media participants perceived not only significant opportunities associated with telemedicine and mHealth but also barriers to overcome to realize these opportunities.

**Conclusions:**

We identified interesting issues in this paper by studying a social media platform in China. Among other things, participants in the selected platform raised concerns about quality and costs associated with the provision of telemedicine and mHealth, despite the new technology’s great potential to address different issues in the Chinese health sector. The methods applied in this paper have some limitations, and the findings may not be generalizable. We have discussed directions for further research.

## Introduction

### Background

In recent years, the use of telemedicine and mHealth has increased rapidly in the People’s Republic of China (hereafter China) [1]. This new technology enables health care providers and patients to meet over the internet and save transportation costs. Research has estimated the Chinese mHealth market to be worth 1.86 billion Chinese Renminbi (RMB), equivalent to US $271 million, during 2012-2013 [2]. By the end of 2017, the market could reach 10 billion RMB (US $1.46 billion) [2].

In this study, we examined a social media platform with a dedicated focus on health information technology and informatics in China. The platform has attracted subscribers from diverse backgrounds: physicians, patients, entrepreneurs, information technology professionals, and other social groups. Exploring the posts from these subscribers allows for understanding the perceptions regarding telemedicine and mHealth in a particular country. Our goal is to utilize the findings from this study to support further research.

### Telemedicine and mHealth in China

First, we provide the definitions of two key terms in this paper. Telemedicine can be defined as provision of medical services from one site to another using electronic communication devices [3,4]. This technology has been developed for several decades. In China, telemedicine was used as early as in the mid-1980s [1], when Chinese physicians consulted patients via telegram; “mHealth” expands telemedicine. It can be defined as “medical and public health practice supported by mobile devices, such as mobile phones, patient monitoring devices, personal digital assistants, and other wireless devices” [5]. mHealth devices expand telemedicine because they enable patients to receive care from health providers without using a desktop computer at a physically fixed location [6-8].

China’s telecommunication infrastructure is quite mature; thus, it can support the delivery of telemedicine and mHealth services for health providers [2,4-5]. Several large telecommunication networks have been actively involved in providing telemedicine services, including International MedioNet of China and the Golden Health Network [5]. While there are more than 940 million mobile phone and app users in China [9], keen competitions among companies have made voice calls and data usage inexpensive for mobile phone subscribers. For instance, many Chinese mobile phone subscribers use the function of text messaging, either synchronously or asynchronously. Mobile phone carriers charge subscribers approximately US $0.015 for sending a text message [10].

Regarding health service provision, there are significant disparities between rural and urban China [11]. Young, highly qualified physicians prefer staying in urban communities [9-13]. To reduce disparities in health services, Chinese government agencies and hospitals are eager to utilize telemedicine and mHealth technology [14-16]. These organizations also believe that elderly patients can benefit from telemedicine and mHealth in various ways [17]. Nonetheless, many Chinese patients and physicians are still skeptical about the new technology [18]. Moreover, for hospitals and clinics, installation and maintenance costs are associated with the provision of telemedicine and mHealth services [17,19].

## Methods

### Social Media Platform Selection

In this exploratory study, we selected a social media platform—HC3i.cn—to examine the perception of telemedicine and mHealth in China. Research has shown that there are different relative advantages of examining “generic” (eg, Facebook or QQ) and “specialized” (eg, patientslikeme.com or Dingxiang Yuan) platforms [20]. Our selected platform is specialized, enabling us to focus on collecting relevant data. The major drawback is that it does not necessarily represent the broad concerns of all health stakeholders in China. We have paid attention to this drawback, however, when drawing conclusions from data collected for this paper.

HC3i.cn was founded in 2010. It is the first internet-based platform that focuses on health informatics, internet-based medicine, and mHealth in China. As of now, it has attracted 150,000 registered users, with a monthly average of 8 million page views. Within the larger HC3i platform, there are more than 30 million posts. They are organized by specific forums; we have examined one of them in this paper, and it is entitled “telemedicine or mHealth” (or “*yuan cheng yi liao or yi dong yi liao* ” in Chinese). The selection is consistent with our research focus in this paper. As of May 16, 2014 (our research cutoff date), this specific forum had 571 threads with 2811 participation posts (ie, each new thread consisted of 2811/571=4.92 posts on average). The earliest thread was initiated on November 21, 2009, and new posts continued to appear on the research cutoff date (May 16, 2014).

Some researchers have pointed out that robots (“bots”) can generate social media content [21]. There are specific algorithms to detect bot activities [21]. Nonetheless, data limitations disallow us to utilize these algorithms. Importantly, bot activities normally do not change the nature of the interactions. They might increase or decrease the magnitude of regular human users’ activities to a certain extent. A full investigation of bot activities in the social media forum under our research will be the subject of a subsequent publication.

### Keyword Analysis

As a text mining technique, we adopted a modified “bag of words” approach [22] to extract keywords in the selected forum. This approach is appropriate for exploratory research. Under this approach, we treat each post in the selected forum as a “document” that consists of individual words. All the posts that we included in the analysis constitute the corpus of documents. Within the corpus, every word is potentially important and can be regarded as a “keyword.” Keyword denotes the importance of a word, defined by frequency or other criteria (eg, a term that leads to many discussions). The frequency criterion indicates the prevalence of a term, which is sometimes called “term frequency (TF) representation.” This approach is straightforward and computationally efficient to operate [22].

We follow several data cleaning steps. If the corpus contains English words, we ignore grammar, word order, sentence structure, and punctuation. In English documents, term normalization steps include changing all words to lower case, so that “iPhone,” “iphone,” “IPHONE” are treated as the same term. Stemming removes suffixes and normalizes tenses and plurals of a word. That is, “play,” “playing,” and “played” are the same. “Stop-words” that are common but have no substantive meaning are also removed. For example, “and,” “the,” “of,” and other prepositions are typically eliminated in frequency representation. For Chinese texts, additional data cleaning steps are necessary [22]. Different from English, Chinese sentences are written in a continuous sequence of characters without the use of delimiters such as the blank space. Besides, individual Chinese characters may or may not constitute a word, so the segmentation of Chinese words needs to be predefined with a dictionary or processed with specific techniques that can distinguish syntactic and semantic units [23].

We applied the “dictionary approach” to identify keywords. Two coders on our research team examined the first post of each of the 571 threads using the word count function in Excel and identified a number of keywords (or categories of them) that have substantive meaning. Notably, not all words that appeared frequently were selected (eg, pronouns, prepositions); only words with a substantive meaning were selected for further analysis.

After analyzing the first posts, we increased the sophistication of keyword identification using an additional criterion. That is, while frequency is concerned with the prevalence of a term, we also considered the sparseness of a term as an additional criterion of importance. Following the text mining literature, a term that occurs in every single document within the entire corpus of documents does not necessarily have any distinguishing power, whereas a term that occurs only in a few documents could have strong distinguishability [24]. The sparseness of a term may be used as a factor to increase the weight of a term’s frequency. The inverse document frequency (IDF) is used in the literature to capture this weight:

IDF(t)=1+log ((Total number of documents)∕(Number of documents containing t))

Combining TF and IDF, we can evaluate each term’s term frequency–inverse document frequency (TFIDF) using the following equation:

TFIDF (t, d) = TF(t, d) * IDF (t)

Where t denotes the term (or keyword) and d denotes the document.

### Qualitative Analysis

To supplement quantitative keyword analysis, we organized the identified keywords into meaningful themes to facilitate interpretations. In the text mining literature, there are quantitative and qualitative approaches to organize keywords into meaningful groups. Clustering is a quantitative technique that considers the coexistence and correlation of multiple terms. Simply put, keywords that are highly correlated are often used together, and they may constitute a theme [25]. An alternative approach is akin to qualitative research [26] and is appropriate for exploratory research. This approach requires the researcher to “code” text data by categorization and interpretations [26]. It is a common technique in qualitative and ethnographic research, which involves elaborate procedures to ensure consistency and reliability [27,28].

Specifically, we modified the hierarchical procedure to code keywords and organize them into themes [26]. First, two coders identified meaningful keywords from the corpus. This step was facilitated by keyword counting, as reported above. Second, each coder independently selected meaningful pairs (or groups) of keywords to generate a theme. The selection required coders to include a note about the coder’s rationale based on knowledge from the literature or experience with the Chinese health sector. Then, the coders were required to crosscheck with each other to reach a consensus and ensure consistency. Based on consensus and practical concerns, the research team determined that 16 prominent keywords with a high frequency of appearance in the selected forum were worthy of greater in-depth analysis and interpretations. These themes facilitated comparisons in the keyword analysis. In the qualitative analysis, the research team further collapsed the themes into 2 higher-level categories in relation to the major research question(s).

## Results

### Keyword Analysis

The number of participation entries to each of these 571 threads—including the first post and all subsequent “replies”—varies, ranging from 0 to 59 as of our research cutoff date. The most popular thread has attracted 59 participation entries. The first post was titled “Education needed: Right now how many level-3A hospitals in China have used telemedicine?” (“Level-3A” indicates the highest rank in an order developed to represent the level of a Chinese hospital’s infrastructure development) [29,30].

Table 1 groups these keywords into 8 themes according to the coding procedures reported above. Thus, we organized the most prominent 16 keywords from 571 threads into 8 themes: (1) Question versus Answer; (2) Hospital versus Clinic; (3) Market versus Company; (4) Doctor versus Nurse; (5) Family versus Patient; (6) iPad versus Tablet; (7) System versus App; and (8) Security versus Caregiving.

Of all first posts, 16.3% (93/571) included a question mark, indicating that many HC3i participants were seeking answers or solutions to some problems; in comparison, 4.9% (28/571) of first posts mentioned “case study,” which were typically discussions on a technical or company solution to problems in implementing telemedicine or mHealth technology. Another intriguing pair was “hospital” and “clinic.” Results suggested that Chinese stakeholders might still see telemedicine and mHealth technology as used primarily within hospitals. While 10.2% (58/571) of first posts mentioned “hospital or inpatient,” only 5.4% (31/571) of first posts mentioned “clinic or outpatient.”

Regarding business opportunities, 3.7% (21/571) of first posts mentioned “market” as opposed to 1.6% (9/571) that mentioned “company.” In terms of health professionals, 3.9% (22/571) of all first posts mentioned “doctor,” but only 1.2% (7/571) mentioned “nurse.” In terms of health service recipients, 1.2% (7/571) of all first posts mentioned “patient” and 0.7% (4/571) mentioned “family.”

**Table 1:** Keywords identified in threads.

Number and themes		Frequency, n (%)^a^
**1**		
	Question	93 (16.3)
	Answer	28 (4.9)
**2**		
	Hospital	58 (10.2)
	Clinic	31 (5.4)
**3**		
	Market	21 (3.7)
	Company	9 (1.6)
**4**		
	Doctor	22 (3.9)
	Nurse	7 (1.2)
**5**		
	Family	4 (0.7)
	Patient	7 (1.2)
**6**		
	iPad	13 (2.3)
	Tablet	1 (1.2)
**7**		
	System	64 (11.2)
	App	18 (3.2)
**8**		
	Security	7 (1.2)
	Caregiving	17 (3.0)

^a^Percentage: Frequency/Number of threads. Number of threads=571.

In terms of technological devices, “iPad” appeared in 2.3% (13/571) of all first posts, whereas “tablet” (participants typically used the word “tablet” to mean an Android-based tablet) appeared in only 1.2% (1/571) of all first posts. A greater percentage of first posts were about how telemedicine and mHealth could work with a health organization’s larger technical “system,” occupying 11.2% (64/571) of all first posts; in contrast, “app” was mentioned only in 3.2% (18/571) of all first posts. Finally, the comparison between “caregiving” and “security” concerns was 3.0% (17/571) versus 1.2% (7/571).

### Term Frequency–Inverse Document Frequency of Keywords From Follow-up Posts

We then examined follow-up posts in the threads generated by the first posts, focusing on threads that had generated at least 20 replies. These replies become the corpus of documents in the subsequent analysis. Using the formula for TFIDF mentioned above, we computed the TFIDF of the same keywords that we used in the previous step to examine the first post. We omitted keywords that either had a lot of missing information or made little sense in the interpretation. Table 2 displays the results.

The corpus of documents consisted of 1977 posts in this analysis. The TF, IDF, and TFIDF of the identified keywords are listed in Table 2, which shows that the list of keywords is generally consistent with those identified in Table 1. TFIDF enables us to recognize the contrast more clearly and facilitate interpretations. First, based on the first 2 keywords, the emphasis on “questions” was about 3 times stronger than that on “answers” (590.3 vs 199.8) in this forum. In terms of utilizing telemedicine, the emphasis was about 14 times stronger on “hospitals” than on “clinics” (660.6 vs 46.5). HC3i participants appeared to pay more attention to how the company can support the development of telemedicine and mHealth than to the market of the new technology, as indicated by a ratio of 8.5 (471.2 vs 55.7) between the keywords “company” and “market.”

**Table 2:** Term frequency–inverse document frequency of selected keywords.

Number and themes		Term frequency–inverse document frequency = (Term frequency × Inverse document frequency)	Term frequency	Inverse document frequency
**1**				
	Question	590.3	150.0	3.9
	Answer	199.8	38.0	5.3
**2**				
	Hospital	660.6	173.0	3.8
	Clinic	46.5	7.0	6.6
**3**				
	Market	55.7	9.0	6.2
	Company	471.2	112.0	4.2
**4**			
	Doctor	458.6	106.0	4.3
	Nurse	340.0	73.0	4.7
**5**				
	Family	28.8	4.0	7.2
	Patient	203.7	39.0	5.2
**6**				
	iPad	331.4	74.0	4.5
	Tablet	433.4	103.0	4.2
**7**				
	System	397.7	91.0	4.4
	App	84.3	14.0	6.0
**8**				
	Security	103.6	18.0	5.8
	Caregiving	305.7	68.0	4.5

In terms of health providers, the emphasis on “doctor” and “nurse” was similar (458.6 vs 340.0). However, in terms of service recipients, the emphasis was 7 times stronger on “patient” than on “family” (203.7 vs 28.8). It did not seem to matter much for HC3i participants whether the device to deliver telemedicine and mHealth was an “iPad” or “tablet” (331.4 vs 433.4). Participants’ concern for the “system” was much stronger than that for the “app,” as indicated by a ratio of 4.7 (397.7 vs 84.3). Finally, there was stronger emphasis on “caregiving and nursing” than on “safety and security,” as indicated by a ratio of 3.0 (305.7/103.6).

### Qualitative Analysis

As mentioned above, we organized the most prominent 16 keywords from 571 threads into 8 themes (see Table 1). These themes often represented how HC3i participants made a practical choice or put greater emphasis between two related items (eg, whether iPad or Android-based tablet is more appropriate for providing mHealth services in China) when they considered telemedicine and mHealth. The following discussions are based on a further collapsing of these themes into 2 higher-level categories regarding the perception of telemedicine and mHealth in China: (1) perceived opportunities and (2) perceived barriers. We selected exemplary quotes and phrases from HC3i participants to support the analysis.

### Perceived Opportunities

In terms of opportunities brought forth by telemedicine and mHealth technology, many HC3i participants believed that the new technology would expand services that can be delivered by health professionals, particularly physicians and nurses. Increasing access to health services for patients and families from underserved areas was mentioned by a number of HC3i participants. One participant commented: “You can now stay at home and shop [online]. [And] won’t [it] be great to stay at home and [still can] ‘see’ a doctor?”

Between “quality” and “cost,” participants paid slightly greater attention to quality. New technological possibilities were believed to lead to new lifestyles to remain healthy. For example, a participant suggested that telemedicine and mobile devices could “digitalize the function of human bodies.” Similarly, other HC3i participants suggested that patients or healthy individuals could utilize wearable technologies to record heartbeat, body temperature, sleep conditions, mood, weight change, and other vital information. Records of this information could help both patients and caregivers better monitor the body, including the recovery progress of individuals suffering from chronic diseases.

In this forum, many participants believed that telemedicine and mHealth technology could help certain companies expand their businesses, but participants were relatively less concerned with the larger market. These participants recognized that telemedicine and mobile devices had a very promising economic prospect if combined with business apps. Several participants pointed out that China—with a population of 1.3 billion—has a huge market for health products. As one participant put it: “Each disease is worth billions [of dollars].” This participant also mentioned that the mobile app Weixin—which has already enrolled millions of Chinese users—could be further explored to build new apps. For this forum participant, “Only if a small proportion of [Weixin] users adopt telemedicine or mobile apps for health purposes, there will be enormous market opportunities [returns].”

### Perceived Barriers

Although excellent opportunities were associated with telemedicine, forum participants raised concerns regarding barriers. First, participants were eager to understand how telemedicine might be compatible with other existing technologies. Some participants saw the use of telemedicine and mHealth technology as primarily for diagnosis and treatment within hospitals. In this sense, telemedicine and mHealth technology could improve health care access to Chinese patients only if they could be admitted to a hospital or registered as a hospital outpatient. However, it is difficult for Chinese patients from some regions to find a hospital in the neighborhood of their residence; thus, there can be strong barriers to be admitted to a hospital or registered as an outpatient [31].

Moreover, some forum participants were skeptical about how health professionals could actually apply telemedicine or mobile devices in practice. Some labeled telemedicine and mobile devices as “an idea only,” “toys for doctors,” or used other unfavorable descriptions. The slightly more optimistic participants called this new technology “a plausible model of health delivery,” but they still remained worried that health professionals might not feel comfortable with different computing interfaces. For them, if physicians and nurses did not fully embrace telemedicine, the delivery of health care cannot be satisfactory. One of these participants wrote: “There is some use with a tablet to provide health care, but mostly for high-end medical applications. For a nurse, this is not really useful. What they [nurses] need is a movable cart for medical devices with remote capabilities, not an entire mHealth system. Many [mHealth systems] are just products of engineers’ imagination.”

Other threads indicated forum participants’ concerns regarding interoperability among telemedicine, mHealth devices, and existing health information technologys. According to these participants, interoperability issues could generate large startup costs to lay the infrastructure for implementing telemedicine; thus, the new technology might not see quick returns to investment. For example, using new mobile devices required barcode scanning, interfacing using radiofrequency identification, existing electronic health records, and the like. One participant was concerned whether a convenient mobile device could be built: “putting everything together makes [the device] ugly [and cumbersome].”

Another group of participants was concerned about standardizing telemedicine and mHealth. In this respect, participants pointed out that the concepts of telemedicine and mHealth are too broad and there is no universal standard to govern this new technology. Among other things, it made it difficult for health professionals and patients to ensure data security. One participant saw the application of telemedicine devices as going between different health care settings—such as between a physician’s office and a patients’ room. When a physician is too busy, he or she could leave the telemedicine-related tablet at insecure places; thus, sensitive information about patients, such as their diagnosis and treatment plans, might fall into inappropriate hands.

Finally, participants raised concerns about the amount and type of human resources needed to support telemedicine devices and systems. For example, some participants had worked in blood banks within hospitals where some mobile devices were used. According to these participants, in such a crowded and busy environment, it was unclear who was accountable for operating the mobile devices properly. The use of these mobile devices was, therefore, prone to frequent errors and could generate undue stress among staff.

## Discussion

Examining social media data is a relatively new methodology in the growing field of health analytics [32-36]. In this paper, we have identified a number of interesting issues from a social media platform on telemedicine, mHealth, and related technology. Several observations are worthy of further investigation: first, many participants of the selected social media platform still saw telemedicine as something used primarily within hospitals [23]. Although some participants mentioned that patients could receive consultation at home by physicians from afar, this did not represent the general view of forum participants. Therefore, developers need to increase the technical soundness of new devices to convince patients and physicians that telemedicine and mHealth can be used “outside” hospitals and that new devices can really deliver the same level of high-quality health care as face-to-face consultations. In future research, it will be useful to compare China with other developed and less developed countries.

As our findings show, there was a strong concern with respect to interoperability among new telemedicine, mHealth, and existing technology. Understandably, health providers did not want to “start everything anew.” Forum participants often linked telemedicine and mHealth to familiar and established technology firms, such as Google, Microsoft, and Apple. Further research can examine whether these firms have a significant market advantage over smaller companies when promoting telemedicine and mHealth products in China [8]. If that were the case, smaller companies, hospitals, and clinics may need to incur significant costs to develop telemedicine and mHealth before they can realize cost savings for patients in the current Chinese market [37,38].

Finally, we acknowledge the limitations of this research; furthermore, the generalizability of our findings may be limited. First, the delimitation of Chinese sentences differs from the English ones in several ways. One approach is to segment the sentence into words in Chinese language processing by character-based sequence labeling. Existing algorithms such as Viterbi might be further explored [20]. Although our approach of utilizing a predefined set of words is feasible, it is not necessarily the most efficient and precise detection method. As mentioned, it is important to examine whether bots are used to generate contents on any social media platform [39], and more sophisticated research is needed in this regard.

## Abbreviations

IDF: inverse document frequency
RMB: Chinese Renminbi
TF: term frequency
TFIDF: term frequency–inverse document frequency
